# Supplementary material for: Antimicrobial Resistance of Staphylococcus aureus Isolated between 2017 and 2022 from Infections at a Tertiary Care Hospital in Romania
Source: Antibiotics (Basel). 2023 May 28;12(6):974. doi: 10.3390/antibiotics12060974 (PMC10294969; doi:10.3390/antibiotics12060974)
Supplement: Supplementary file 1 [file antibiotics-12-00974-s001.zip › antibiotics-2383002-supplementary.pdf]

Table S1. Romania GP 1 EUCAST.

	1	2	3	4	5	6	7	8	9	10	11	12
A	PEN 0.0625	PEN 0.125	PEN 0.25	PEN 0.5	PEN 1	PEN 2	AMP 0.5	AMP 1	AMP 2	AMP 4	AMP 8	SNH 512
B	OXA 0.25	OXA 0.5	OXA 1	OXA 2	OXA 5	CRO 0.5	CRO 1	CRO 2	CRO 1	CFL 2	COX 4	COX 8
C	CIP 0.25	CIP 0.5	CIP 1	CIP 2	CIP 4	LEV 1	LEV 2	LEV 4	MOX 0.25	MOX 0.5	TOB 1	TOB 2
D	ERY 0.25	ERY 0.5	ERY 1	ERY 2	ERY 4	CLI 0.25	CLI 0.5	CLI 1	ERC 4/0.5	GEN 1	GEN 2	GNH 128
E	VAN 1	VAN 2	VAN 4	VAN 8	TPL 1	TPL 2	TLP 4	TLP 8	DPT 1	DPT 2	AMK 8	AMK 16
F	DOX 1	DOX 2	DOX 4	MNO 0.5	MNO 1	MNO 2	TET 1	TET 2	TET 4	TGC 0.25	TGC 0.5	TGC 1
G	T/S 0.03125 / 0.59375	T/S 0.0625/ 1.1875	T/S 0.125/ 2.375	T/S 0.5/9.5	T/S 1/19	T/S 2/38	T/S 4/76	LIZ 1	LIZ 2	LIZ 4	NFT 64	NGC
H	RAM 0.0625	RAM 0.125	RAM 0.25	RAM 0.5	RAM 1	CMP 8	CMP 16	FOS 32	FOS 64	FUS 1	FUS 2	GC

LEGEND			
**AMK**	Amikacin	**LEV**	Levofloxacin
**AMP**	Ampicillin	**LIZ**	Linezolid
**COX**	Cefoxitin	**MNO**	Minocycline
**CFL**	Ceftaroline	**MOX**	Moxifloxacin
**CRO**	Ceftriaxone	**NGC**	Negative control
**CMP**	Chloramphenicol	**NFT**	Nitrofurantoin
**CIP**	Ciprofloxacin	**OXA**	Oxacillin

**CLI**	Clindamycin	**PEN**	Penicillin G
**DPT**	Daptomycin	**RAM**	Rifampicin
**DOX**	Doxycycline	**SNH**	Streptomycin high level
**ERY**	Erythromycin	**TPL**	Teicoplanin
**ERC**	Erythromycin/Clindamycin	**TET**	Tetracycline
**FOS**	Fosfomycin	**TGC**	Tigecycline
**FUS**	Fusidic acid	**TOB**	Tobramycin
**GEN**	Gentamicin	**T/S**	Trimethoprim/Sulfamethoxazole
**GNH**	Gentamicin high level	**VAN**	Vancomycin
**GC**	Growth control		

TableS2. Romania GP 2 EUCAST

	1	2	3	4	5	6	7	8	9	10	11	12
A	PEN 0.0625	PEN 0.125	PEN 0.25	PEN 0,5	PEN 1	PEN 2	AMP 0.5	AMP 1	AMP 2	AMP 4	AMP 8	SNH 512
B	OXA 0.25	OXA 0.5	OXA 1	OXA 2	CRO 0.125	CRO 0.25	CRO 0.5	CRO 1	CRO 2	CFL 0.5	CFL 1	CFL 2
C	CIP 0.0625	CIP 0.125	CIP 0.25	CIP 0.5	CIP 1	CIP 2	CIP 4	LEV 1	LEV 2	LEV 4	TOB 1	GEN 1
D	ERY 0.25	ERY 0.5	ERY 1	ERY 2	CLI 0.25	CLI 0.5	ERC 4/0.5	MOX 0.25	MOX 0.5	MOX 1	AMK 8	GNH 128
E	VAN 1	VAN 2	VAN 4	TPL 1	TPL 2	TPL 4	DPT 1	COX 4	COX 8	FEP 0.5	FEP 1	FEP 2
F	DOX 0.25	DOX 0.5	DOX 1	DOX 2	MNO 0.5	MNO 1	TET 1	TET 2	TGC 0.125	TGC 0.25	TGC 0.5	NFT 64
G	T/S 0.5/9.5	T/S 1/19	T/S 2/38	T/S 4/76	LIZ 1	LIZ 2	LIZ 4	MER 0.25	MER 0.5	MER 1	MER 2	NGC
H	RAM 0.0625	RAM 0.125	RAM 0.25	RAM 0.5	RAM 1	CMP 2	CMP 8	FOS 32	FUS 1	DAL 0.125	DAL 0.25	GC

LEGEND			
**AMK**	Amikacin	**LEV**	Levofloxacin
**AMP**	Ampicillin	**LIZ**	Linezolid
**COX**	Cefoxitin	**MER**	Meropenem
**CFL**	Ceftaroline .	**MNO**	Minocycline
**CRO**	Ceftriaxone	**MOX**	Moxifloxacin
**CMP**	Chloramphenicol	**NGC**	Negative control
**CIP**	Ciprofloxacin	**NFT**	Nitrofurantoin

**CLI**	Clindamycin	**OXA**	Oxacillin
**DAL**	Dalbavancin	**PEN**	Penicillin G
**DPT**	Daptomycin	**RAM**	Rifampicin
**DOX**	Doxycycline	**SNH**	Streptomycin high level
**ERY**	Erythromycin	**TPL**	Teicoplanin
**ERC**	Erythromycin/Clindamycin	**TET**	Tetracycline
**FEP**	Cefepim	**TGC**	Tigecycline
**FOS**	Fosfomycin	**TOB**	Tobramycin
**FUS**	Fusidic acid	**T/S**	Trimethoprim/Sulfamethoxazole
**GEN**	Gentamicin	**VAN**	Vancomycin
**GNH**	Gentamicin high level		
**GC**	Growth control		

Table S3. Romania GP 3 EUCAST

	1	2	3	4	5	6	7	8	9	10	11	12
A	PEN<br>0.0625	PEN<br>0.125	PEN<br>0.25	PEN<br>0,5	PEN<br>1	PEN<br>2	AMP<br>0.5	AMP<br>1	AMP<br>2	AMP<br>4	AMP<br>8	SNH<br>512
B	OXA<br>0.25	OXA<br>0.5	OXA<br>1	OXA<br>2	CRO<br>0.125	CRO<br>0.25	CRO<br>0.5	CRO<br>1	CRO<br>2	CFL<br>0.5	CFL<br>1	CFL<br>2
C	CIP<br>0.0625	CIP<br>0.5	CIP<br>1	CIP<br>2	CIP<br>4	LEV<br>1	LEV<br>2	LEV<br>4	AMK<br>16	AMK<br>32	GEN<br>2	GEN<br>4
D	ERY<br>0.25	ERY<br>0.5	ERY<br>1	ERY<br>2	CLI<br>0.25	CLI<br>0.5	ERC<br>4/0.5	MOX<br>0.25	MOX<br>0.5	MOX<br>1	TOB<br>2	GNH<br>128
E	VAN<br>1	VAN<br>2	VAN<br>4	TPL<br>1	TPL<br>2	TPL<br>4	DPT<br>1	COX<br>4	COX<br>8	CEP<br>0.5	CEP<br>1	CEP<br>2
F	DOX<br>0.25	DOX<br>0.5	DOX<br>1	DOX<br>2	MNO<br>0.5	MNO<br>1	TET<br>1	TET<br>2	TGC<br>0.125	TGC<br>0.25	TGC<br>0.5	NFT<br>64
G	T/S<br>0.5/9.5	T/S<br>1/19	T/S<br>2/38	T/S<br>4/76	LIZ<br>1	LIZ<br>2	LIZ<br>4	MER<br>0.25	MER<br>0.5	MER<br>1	MER<br>2	NGC
H	RAM<br>0.0625	RAM<br>0.125	RAM<br>0.25	RAM<br>0.5	RAM<br>1	CMP<br>2	CMP<br>8	FOS<br>32	FUS<br>1	DVA*<br>0.125	DVA*<br>0.25	GC

LEGEND			
**AMK**	Amikacin	**LEV**	Levofloxacin
**AMP**	Ampicillin	**LIZ**	Linezolid
**CEP**	Cefepim	**MER**	Meropenem
**COX**	Cefoxitin	**MNO**	Minocycline
**CFL**	Ceftaroline	**MOX**	Moxifloxacin
**CRO**	Ceftriaxone	**NGC**	Negative control
**CMP**	Chloramphenicol	**NFT**	Nitrofurantoin

**CIP**	Ciprofloxacin	**OXA**	Oxacillin
**CLI**	Clindamycin	**PEN**	Penicillin G
**DVA***	Dalbavacin (RUO)	**RAM**	Rifampicin
**DPT**	Daptomycin	**SNH**	Streptomycin high level
**DOX**	Doxycycline	**TPL**	Teicoplanin
**ERY**	Erythromycin	**TET**	Tetracycline
**ERC**	Erythromycin/Clindamycin	**TGC**	Tigecycline
**FOS**	Fosfomycin	**TOB**	Tobramycin
**FUS**	Fusidic acid	**T/S**	Trimethoprim/Sulfamethoxazole
**GEN**	Gentamicin	**VAN**	Vancomycin
**GNH**	Gentamicin high level	**GC**	Growth control

Table S4. VITEK AST-P592 Card

Antibiotics		Concentrations	Antibiotics		Concentrations
**AM**	Ampicillin	0.5; 4; 8; 32.	**RA**	Rifampicin	0.25; 0.5; 2.
**CIP**	Ciprofloxacin	1; 2; 4.	**TE**	Tetracycline	0.5; 1; 2.
**CM**	Clindamycin	0.5; 1; 2.	**TEC**	Teicoplanin	1; 4; 8.
**HLG**	Gentamicin high level	500	**TGC**	Tigecycline	0.25; 0.5; 1.
**HLS**	Streptomycin high level	1000	**SXT**	Trimethoprim/Sulfamethoxazole	8/152; 16/304; 32/608.
**ICR**	Inducible Clindamycin Resistance	Negative/Positive	**VA**	Vancomycin	1; 2; 4; 8; 16.
**IPM**	Imipenem	2; 4; 8.			
**LNZ**	Linezolid	0.5; 1; 2.			
**MXF**	Moxifloxacin	0.25; 2; 8.			
**OX1**	Oxacillin	0.5; 1; 2.			
**OXSF**	Cefoxitin Screen	Negative/Positive			
**P**	Benzylpenicillin	0.125; 0.25; 1.			
